# Spider Silk-CBD-Cellulose Nanocrystal Composites: Mechanism of Assembly

**DOI:** 10.3390/ijms17091573

**Published:** 2016-09-18

**Authors:** Sigal Meirovitch, Zvi Shtein, Tal Ben-Shalom, Shaul Lapidot, Carmen Tamburu, Xiao Hu, Jonathan A. Kluge, Uri Raviv, David L. Kaplan, Oded Shoseyov

**Affiliations:** 1 The Robert H. Smith Institute of Plant Sciences and Genetics in Agriculture, Robert H. Smith Faculty of Agriculture, Food and Environment, The Hebrew University of Jerusalem, P.O. Box 12, Rehovot 76100, Israel; sigal.meirovitch@mail.huji.ac.il (S.M.); tsvika.shtein@gmail.com (Z.S.); talbs87@gmail.com (T.B.-S.); shaul@melodea.eu (S.L.); 2 The Institute of Chemistry, The Hebrew University of Jerusalem, Edmond J. Safra Campus, Givat Ram, Jerusalem 91904, Israel; carmen.tamburu@mail.huji.ac.il (C.T.); uri.raviv@mail.huji.ac.il (U.R.); 3 Department of Biomedical Engineering, 4 Colby Street, Tufts University, Medford, MA 02155, USA; hu@rowan.edu (X.H.); jonathan.kluge@tufts.edu (J.A.K.); david.kaplan@tufts.edu (D.L.K.); 4 Department of Physics and Astronomy, Rowan University, Glassboro, NJ 08028, USA

**Keywords:** spider silk, cellulose nanocrystals, cellulose binding domain, nanocomposite, biomaterials

## Abstract

The fabrication of cellulose-spider silk bio-nanocomposites comprised of cellulose nanocrystals (CNCs) and recombinant spider silk protein fused to a cellulose binding domain (CBD) is described. Silk-CBD successfully binds cellulose, and unlike recombinant silk alone, silk-CBD self-assembles into microfibrils even in the absence of CNCs. Silk-CBD-CNC composite sponges and films show changes in internal structure and CNC alignment related to the addition of silk-CBD. The silk-CBD sponges exhibit improved thermal and structural characteristics in comparison to control recombinant spider silk sponges. The glass transition temperature (*Tg*) of the silk-CBD sponge was higher than the control silk sponge and similar to native dragline spider silk fibers. Gel filtration analysis, dynamic light scattering (DLS), small angle X-ray scattering (SAXS) and cryo-transmission electron microscopy (TEM) indicated that silk-CBD, but not the recombinant silk control, formed a nematic liquid crystalline phase similar to that observed in native spider silk during the silk spinning process. Silk-CBD microfibrils spontaneously formed in solution upon ultrasonication. We suggest a model for silk-CBD assembly that implicates CBD in the central role of driving the dimerization of spider silk monomers, a process essential to the molecular assembly of spider-silk nanofibers and silk-CNC composites.

## 1. Introduction

Silks are produced by a variety of insects and spiders and some spiders spin as many as seven different kinds of silks, each tailored to fulfill a certain biological function [1]. Dragline silk, used as the safety line and as the frame thread of the spider’s web, is composed of two proteins, each with a long repetitive sequence flanked by nonrepetitive amino and carboxy termini [2,3]. The repetitive sequence is characterized by stretches of poly-alanine domains that are interrupted by glycine-rich repeats. The poly-alanine domains form β-sheet crystals, whereas the glycine-rich repeats form less crystalline segments [4,5,6]. The interplay between the hard crystalline domains and the less crystalline regions gives rise to the extraordinary properties of the silk [7,8]. Dragline silk has unique toughness and higher tensile energy to break than any other common natural or artificial material [9]. Its strength to weight ratio is five times stronger than steel and three times tougher than top quality man-made Kevlar fiber. Due to its superb mechanical properties, dragline silk has been the focus of intense research efforts [1,10].

Despite the extensive knowledge that has been gained regarding the structure and properties of dragline silk, its production remains challenging. In contrast to silkworm-silk, the isolation of large quantities of silk from spiders is not feasible. Spiders produce silk in small quantities, and their territorial behavior prevents large numbers from being raised in a confined space [11]. Therefore, production of spider silk proteins through recombinant DNA techniques has been the primary path pursued by researchers. Silk-encoding genes have been cloned and expressed in a variety of heterologous hosts [12,13,14,15], which have allowed for production of laboratory scale quantities of silk-like protein powders. Yet, with few exceptions [16,17], the material properties of these cloned silks are inferior to their native counterparts. One limitation has been the lack of molecular order in the recombinant silk proteins and their tendency to aggregate in vitro, bypassing the native refolding and assembly processes [10]. Assembly of silk proteins into a solid silk fiber is extremely complex, and replication of the spider’s native spinning process is a major challenge in laboratory settings [18,19]. The current work addresses this challenge using a different strategy—the production of spider silk CNC bio-composites. The technique relies on the specific binding capacity of cellulose binding domains (CBDs) to CNCs and the structural properties of CNCs.

Cellulose is a product of biosynthesis from plants, animals, or bacteria, while “cellulose nanocrystals” refer to cellulosic extracts or processed materials, having defined nano-scale structural dimensions [20]. CNCs are exciting biomaterials that are relevant to a number of potential applications, including polymer nanocomposites, transparent films and hydrogels. CNCs are mainly produced by acid hydrolysis/heat controlled techniques, with sulfuric acid being the most utilized acid. The crystal extraction from cellulose involves hydrolysis of amorphous cellulose regions, resulting in highly crystalline particles with dimensions of 5–20 nm in width and 100–500 nm in length for plant source CNCs [21]. The mechanical properties of CNCs is impressive with the Young’s modulus and tensile strength of a single crystal reported to be as high as 150 and 10 GPa, respectively, which make them useful for the reinforcement of polymers. In addition, the rod-like shape of the particles leads to concentration-dependent liquid crystalline self-assembly behavior [22]. Noishiki et al. [23] found that CNC-native silkworm silk films had breaking strengths and ductility about five times greater than those of the constituent materials. The authors attributed these improvements to the flat and ordered surfaces of CNCs, which served as a template for the assembly of silk β-sheets, a process that usually requires shear and elongation stress.

In nature, cellulose is degraded by the concerted actions of a number of bacterial and fungal organisms, initiated by cellulolytic enzyme(s) or microorganisms that can bind cellulose substrates. The CBD, a separate, nonhydrolytic component, mediates this binding. CBDs have been cloned from different organisms such as *Clostridium cellulovorans* and *Cellulomonas fimi* [24,25], and enable adhesion of the water-soluble enzyme to the soluble or insoluble substrate, by bringing the catalytic module into prolonged and intimate contact with the cellulose surface [26,27].

In the present study, we compare synthetic 15-monomer-long dragline spider silk derived from the native sequence of MaSp1 of *Nephila clavipes*, referred to as “silk”, and the synthetic 15-monomer with CBD fused to its 3′ termini, referred to as “silk-CBD”. Unlike silk, upon sonication, silk-CBD dimerizes and these dimers assemble in situ into microfibrils; this mechanism appears analogous to the formation of silk fibrils in nature, which proceeds via the C-termini. Furthermore, the effects of silk-CBD dimerization upon the mechanisms of CNC self-assembly of the CNCs in sponges and films are explored. The general motivation for composites made from silk and CNCs is to produce materials with characteristics reflective of both components; for instance, silk-cellulose composites may provide a strength and toughness profile that surpasses either component. In this work, we observed that silk-CBD specifically binds CNCs and confers molecular order which is different from that of either the silk proteins or CNCs. Silk-CBD-CNC composite materials may be useful in a variety of medical and industrial applications, and this preliminary research is a necessary step toward the end goal of harnessing the attractive properties of the components into a composite with superior properties.

## 2. Results and Discussion

### 2.1. Protein Expression and Purification

**Figure 1:**
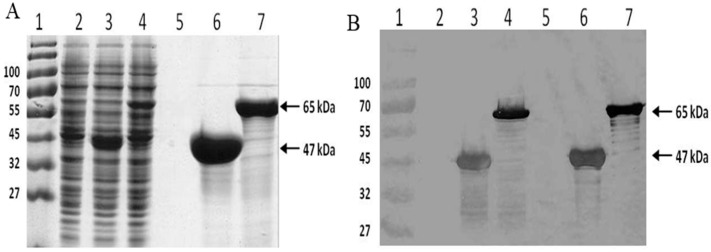
Expression and purification of silk and silk-CBD. SDS-PAGE of soluble *E. coli* proteins, stained with Coomassie blue (**A**); and Western blot analysis (**B**) using an anti-HIS antibody. Lane 1, molecular weight marker; lane 2, total protein of the control bacteria (*E. coli* transformed with an empty vector); lane 3, total protein of silk expressing bacteria; lane 4, total protein of silk-CBD expressing bacteria; lane 5, control sample (empty vector) after Ni-NTA purification; lane 6, purified silk protein; and lane 7, purified silk-CBD fusion protein.

The synthetic spider silk and the fusion spider silk-CBD genes were successfully expressed, and the resultant proteins were purified from *E. coli* using Ni-NTA chromatography. The pure protein yields for the expression of silk (47 kDa) and silk-CBD (65 kDa) were 60 and 40 mg/L, respectively (Figure 1).

### 2.2. Quantitative Cellulose Binding Assay

**Figure 2:**
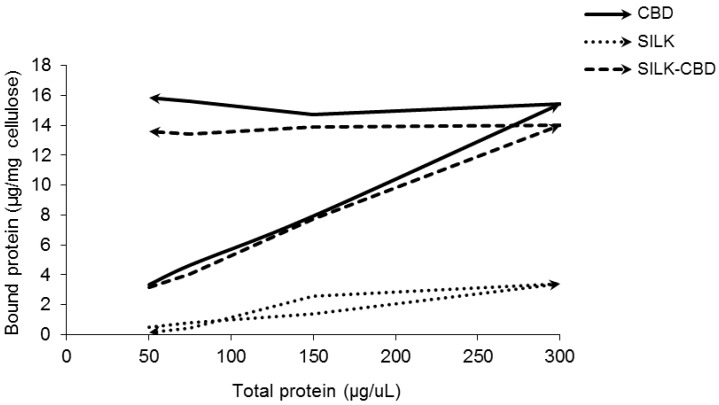
Adsorption/desorption isotherms. CBD (solid line), silk (dotted line), silk-CBD (dashed line), at different concentrations were allowed to adsorb to cellulose (Sigmacell 20) to the point of equilibrium. After equilibrium was reached, the highest protein concentration (1.2 mg/mL) to cellulose mixture was diluted to allow desorption.

In order to characterize the binding capacity of silk-CBD to cellulose, adsorption/desorption experiments were conducted. Adsorption/desorption experiments are commonly done to test the apparent irreversible adsorption of CBD to cellulose. A reversible adsorption process is defined when the variables characterizing the state of the system return to the same values in the reverse order during the desorption stage. Therefore, in a reversible adsorption process, the ascending branch (increasing protein concentration in the solution) and the descending branch (decreasing protein concentration in the solution) of the isotherm overlap. Reversible adsorption was seen for the purified silk protein in solution with cellulose (Figure 2) due to the mechanism of protein adsorption at solid/liquid interfaces [28]. In contrast, irreversible binding was observed for both CBD and silk-CBD as evident from their non-overlapping adsorption isotherms.

### 2.3. Composite CNC/Spider Silk Sponges

Spider silk/CNC composite sponge formation was done as previously described [29,30,31,32]. Purified, concentrated spider silk protein was mixed with a CNC suspension and then sonicated. This procedure has a two-fold effect; in addition to homogeneous dispersion of the CNCs, the sonication process induces annealing of spider silk proteins by accelerating formation of physical cross-links, such as initial chain interactions related to β-sheet formation [33,34]. After sonication, three-dimensional porous structures (i.e., sponges) were generated via freeze drying.

**Figure 3:**
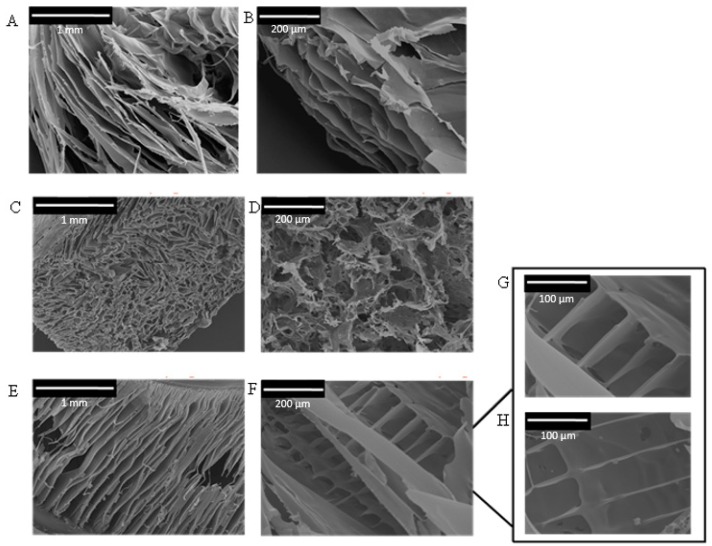
SEM pictures of silk, silk-CBD, and composite silk-CBD-CNC sponges. (**A**,**B**) CNC sponge; (**C**) 100% silk sponge; (**D**) silk-CNC composite sponge (75% silk and 25% CNC); (**E**) 100% silk-CBD sponge; and (**F**–**H**) silk-CBD-CNC composite sponge (75% silk-CBD and 25% CNC) on a magnified scale.

SEM pictures of the resulting sponges showed that pore architecture and alignment differed between the silk-CBD and control silk sponges (Figure 3). Silk sponges had 30–100 μm pores (Figure 3C) of irregular shape and with no particular orientation, very similar to the CNC-silk composites (Figure 3D). Silk-CBD sponges featured 300–500 μm leaf-shaped pores aligned in a relatively consistent direction (Figure 3E). Similar characteristics were observed in sponges from native silkworm silk produced using the same conditions applied here, which were attributed to the parallel arrangements of silk fibroin crystal flakes [30]. The composite silk-CBD-CNC sponges possessed ~100 μm structurally aligned pores (Figure 3F–H).

**Figure 4:**
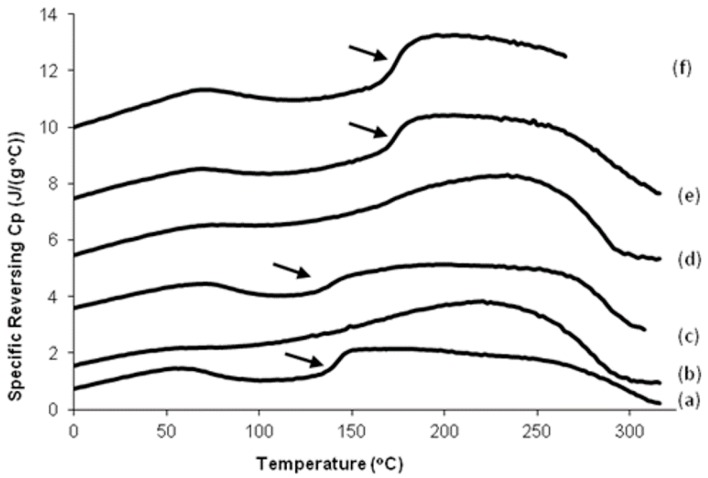
DSC analysis of silk and silk-CBD sponges. Reverse heat flow vs. temperature during TMDSC scanning of silk and silk-CBD sponges at 2 °C/min. (**a**) 100% silk sponge; (**b**) 25% silk/75% CNC sponge; (**c**) 75% silk/25% CNC sponge; (**d**) 25% silk-CBD/75% CNC sponge; (**e**) 75% silk-CBD/25% CNC sponge; and (**f**) 100% silk-CBD sponge. The arrows indicate the glass transition temperatures.

The glass transition and degradation temperatures of different silk and silk-CBD sponges, as determined by TMDSC analysis, are shown in Figure 4 and Table 1. DSC analysis of the 100% silk and silk-CBD sponges gave *Tg* values of 140 and 172 °C, respectively, and degradation temperatures of 279 and 283 °C, respectively. Interestingly, the *Tg* and degradation temperatures of the 100% silk-CBD sponge were similar to those reported for natural silkworm silk and *Nephilia clavipes* dragline silk fibers [35,36,37]. The changes seen in the *Tg*, characteristic of the amorphous domains in amorphous or semi-crystalline materials, such as silks, result from increased chain interactions. The stronger the interactions between the chains, the higher the temperature required to induce a phase transition. The lower *Tg* of the 100% silk sponge may be due to a more disordered structure, as seen in the SEM figures. The elevation in the *Tg* of the 25% silk/75% CNC sponge is likely related to the significant presence of CNCs, whose crystal surfaces may serve as a template/nucleation site for the assembly of silk β-sheets, as seen in the silkworm silk-CNC composite films by Noishiki et al. [23]. As for the elevated *Tg* of the silk-CBD sponges, it has been well established that CBDs form types of dimers in solution [38,39], and this dimerization factor likely also plays a role.

### 2.4. Composite CNC/Spider Silk Films

**Figure 5:**
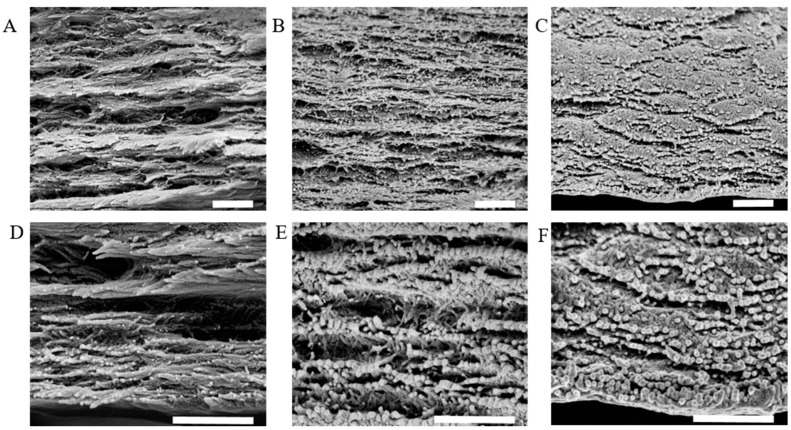
SEM images of CNC film cross-sections (**A**,**D**); silk-CBD-CNC composite film with 1:10 weight ratio (**B**,**E**); and composite films with 1:5 weight ratio (**C**,**F**). The upper images are at 100,000× magnification and the bottom images at 200,000×. (Scale bar = 500 nm).

**Figure 6:**
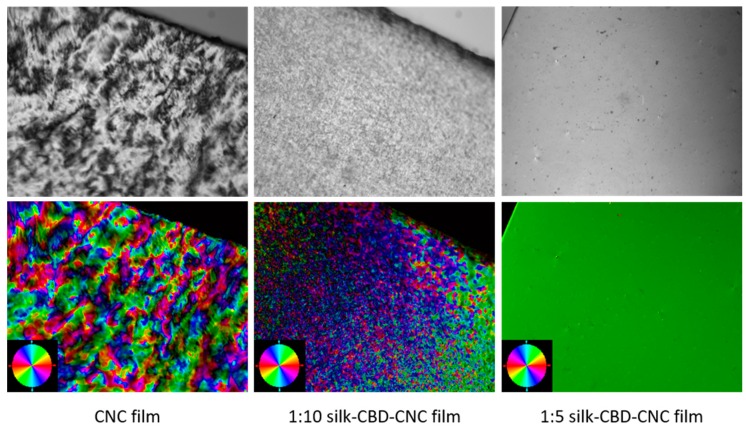
Top images present POM of CNC and silk-CBD-CNC composite films with increasing amounts of silk-CBD. Bottom images show the corresponding LC-PolScope analysis performed on the POM image. The color wheel in the corner presents the orientation of the sample alignment (20× magnification).

**Figure 7:**
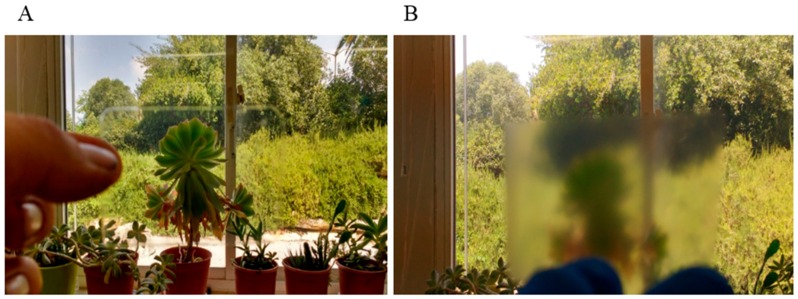
Qualitative comparison of transparency of CNC films compared to silk-CBD CNC 1:10 composite films. (**A**) 1:10 composite silk-CBD-CNC film; (**B**) CNC film.

Films of silk-CBD and CNCs were prepared in order to further investigate the effects silk-CBD on the materials and the role of dimerization. Similar to the sponge results presented in Figure 3, SEM cross-sectional images of CNC and silk-CBD-CNC films at mass ratios of 1:5 and 1:10 (Figure 5) show differences in film morphology related to the presence and amount of silk-CBD. The CNC film shows a typical layered morphology, whereas the composite silk-CBD-CNC films appear to be more aligned and dense. Film alignment was explored using a polarized optical microscopy (POM) system equipped with an image processing module. The top images in Figure 6 shows POM images of CNC and silk-CBD-CNC composite films, where the bright and dark regions typically indicate ordered and disordered areas in the films, respectively. The bottom images in Figure 5 show the processed birefringence images. The CNC films show the multi-domain order typical to CNC films, whereas the silk-CBD films show uniform alignment. The Abrio 2.2 software (CRi, Woburn, MA, USA) provides a vector overlay tool to analyze the processed image, where the vector azimuth is measured, and the standard deviation (SD) of the vector direction gives an approximation of the degree of alignment uniformity. An average of twenty measurements per sample (same size area) and corresponding SD values were calculated; an SD of 44.25 was obtained for CNC films, 13.58 for 1:10 silk-CBD-CNC films and 1.33 for 1:5 silk-CBD-CNC films. These SD values indicate improved sample alignment with increasing silk-CBD content. Furthermore, we qualitatively observed that the films appeared more transparent when silk-CBD was present (Figure 7). This may be due to the particular approach to film formation used in this work (i.e., CNC formulations cast onto hydrophobic surfaces) or may be related to the dispersion/self-assembly of the particles in the films.

### 2.5. Spider Silk-CBD Fusion Protein Assembly

To summarize, interesting behavior is observed in the composite materials when silk-CBD is used, including an elevation in the *Tg*, differences in internal structure and morphology, and differences in alignment in the films. As mentioned above, CBD dimerization may play a role in the observed *Tg* elevation and in driving the differences observed in alignment. Gel filtration and dynamic light scattering (DLS) and small angle X-ray scattering (SAXS) analyses were conducted in order to better understand this process and whether it affects the properties of the composite materials presented in this work.

**Figure 8:**
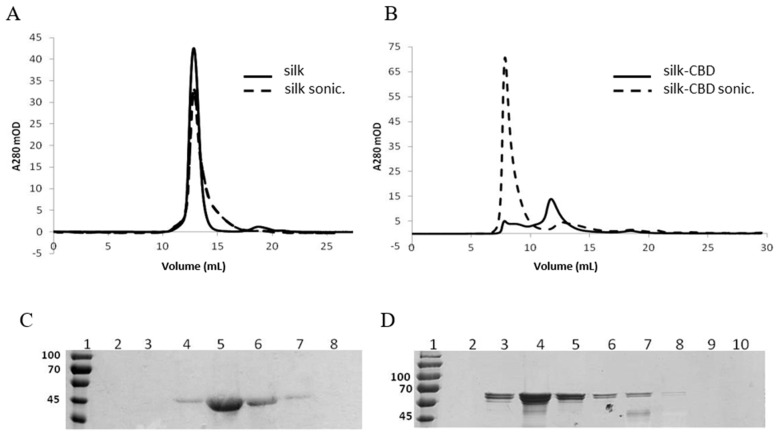
Gel filtration and DLS of silk and silk-CBD samples before and after sonication (sonic.). Representative gel of silk (**A**); and silk-CBD (**B**) protein samples before (solid line) and after sonication (dashed line); SDS PAGE analysis of gel filtration fractions of sonicated silk (**C**); and silk-CBD (**D**) samples.

Protein solutions analyzed by gel filtration and DLS before and after sonication indicated increased molecular weights and diameters of silk-CBD assemblies upon sonication, compared to the control silk protein (Figure 8). The pure silk solution was eluted with a peak corresponding to 140 kDa (Figure 8A), whereas the silk-CBD was eluted as two major peaks, one eluted into the void volume, and the second eluted as a 230 kDa protein, which may correspond to a dimer. After sonication, most of the silk-CBD protein was eluted into the void volume (Figure 8B). The calculated molecular weights of the silk and silk-CBD proteins are 47 and 65 kDa, respectively. SDS-PAGE analysis under denaturing conditions supported the molecular weight calculations (Figure 8C,D). As reported previously, proteins rich in glycine and alanine can migrate anomalously during gel filtration, often resulting in overestimation of the protein molecular weight [40]. In addition, the gel filtration was performed under non-denaturing conditions and silk polypeptides may adopt a rod-like elongated configuration, leading to size overestimation, compared to globular protein standards. It has been established that spider silk proteins form disulfide-bridged homodimers [41,42,43,44]. Gel filtration of the native proteins under reducing conditions revealed 260–320 kDa protein monomers. In the absence of the reducing agent, a shift in the molecular mass to 420–480 kDa was observed, less than a two-fold gain in molecular weight [41]. Dimer formation likely leads to changes in the protein conformation, resulting in modified protein migration along the column, as compared to the monomer form. The silk-CBD dimer may act as a nucleation site for higher molecular weight assemblies, which were eluted into the void volume, even before sample sonication.

After sonication, an increase in the proportion of higher molecular weight silk assemblies was observed, similar to that previously described for native silkworm silk proteins subjected to sonication [33]. As demonstrated by DLS (Table 2), both un-sonicated and sonicated silk formed homogeneous solutions with 3–4 nm diameter particles, whereas un-sonicated silk-CBD had 55 and 260 nm diameter particles, which increased in size to 96 nm and 2 μm particles upon sonication.

### 2.6. Small Angle X-ray Scattering (SAXS) of Solutions of Silk and Silk-CBD

**Figure 9:**
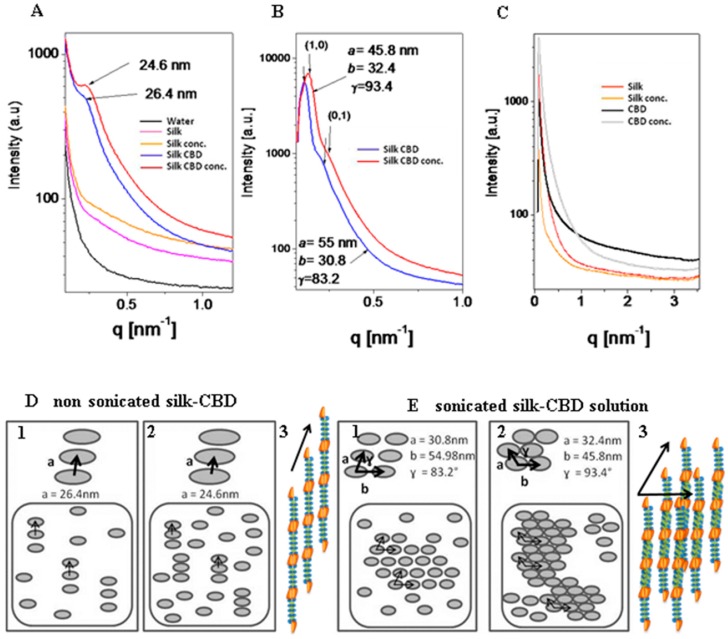
(**A**–**C**) Radially integrated solution small-angle X-ray scattering intensity from silk and silk-CBD non-concentrated and concentrated (conc.) samples before (**A**) and after sonication (**B**,**C**); (**D**,**E**) Schematic illustration of the silk-CBD hierarchical order in non-concentrated (**D1**,**E1**) and concentrated (**D2**,**E2**) samples, before (**D**) and after sonication (**E**). Models of the structures of self-assembled silk-CBD subunits before (**D3**) and after (**E3**) sonication. The orange units represent the CBD moiety and the green and blue units represent the crystalline domains and the less crystalline regions of the silk monomer, respectively.

SAXS measurements of the silk and silk-CBD solutions analyzed before and after sonication (Figure 9) demonstrated a form factor closely matching that of infinitely long rods. The silk samples released a very weak signal with apparently no structurally ordered subunits formed in solution, neither before (Figure 9A) nor after (Figure 9C) sonication. In the case of un-sonicated silk-CBD, the single and rather broad correlation peak (Figure 9A) indicated a nematic liquid crystalline phase, with a correlation distance of 26.4 nm and a domain size of approximately 80 nm. In other words, three subunits are in positional correlation, and the rod center-to-center distance is 26.4 nm (Figure 9D1). After sample concentration (Figure 9A), the peak intensity increased and the correlation distance decreased to 24.6 nm. Namely, the nematic phase was denser and more subunits were in positional correlation with one another (Figure 9D2). In nature, the highly concentrated spider protein dope, much like that of the silkworm, is liquid crystalline, where the main silk protein constituent is likely to assume a compact rod-like conformation. This conformation enables silk protein processing at high concentrations. Specifically, the molecules seem to form a nematic phase in the spider gland and duct, with the long axes of neighboring molecules aligned approximately parallel to one another. Liquid crystallinity offers desirable properties, such as efficient spinning of molecules as large as silk proteins, by allowing the viscous silk protein solution to slowly flow through the storage sac and duct as complex alignment patterns are formed [19]. After sonication (Figure 9B), the silk-CBD correlation peaks were fitted to a two-dimensional oblique lattice with three subunits along the a-axis, forming a rod center-to-center distance of 30.8 nm and three subunits along the b-axis, forming a rod center-to-center distance of 54.98 nm. The alignment angle between the two-dimensional subunits was γ = 83.2° (Figure 9E1); sonication resulted in the addition of subunit interactions, which led to their two-dimensional alignment. After concentrating the solution (Figure 9B), the lattice parameters changed to a = 32.4 nm, b = 45.8 nm and γ = 93.4°, and namely, more subunits were in correlation with one another (Figure 9E2).

### 2.7. Cryo-Transmission Electron Microscopy (Cryo-TEM)

**Figure 10:**
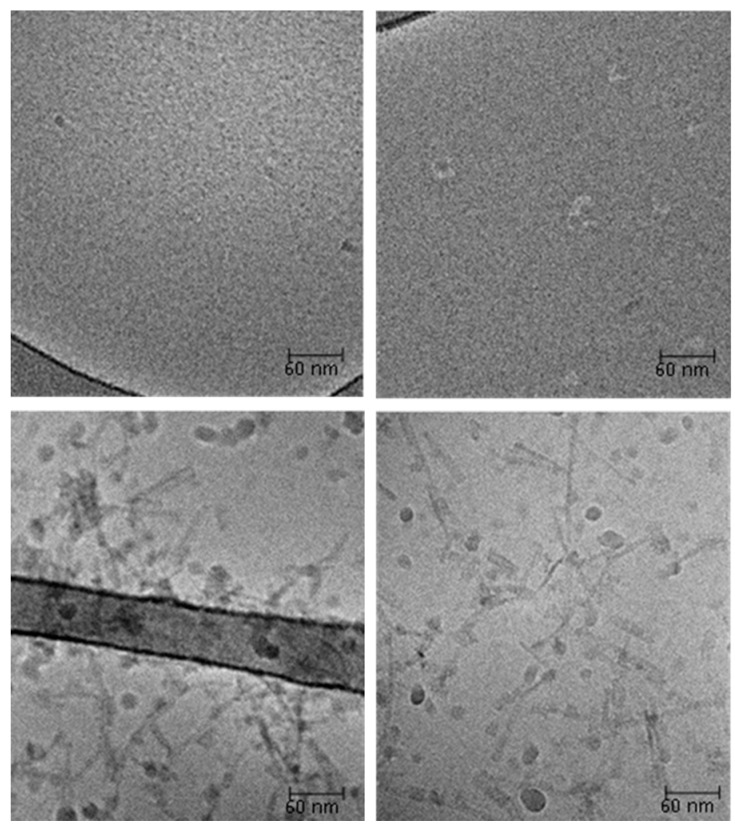
Cryo-transmission electron microscopy (cryo-TEM) images of sonicated solutions of silk (**upper pictures**) and silk-CBD (**bottom pictures**). Scale bar is 60 nm.

Cryo-transmission electron microscopy (cryo-TEM) images of sonicated protein samples support the gel filtration, DLS and SAXS results (Figure 10). After sonication, the silk protein did not form any orderly structures (Figure 10 upper images), whereas silk-CBD formed 100–200 nm long fibers/microfibrils (Figure 10 bottom images). The liquid crystals and the longer structures revealed by SAXS and DLS were not detectable by this method, as they form very thick layers that are removed from the grid when preparing the thin film (<100 nm thick) required for cryo-TEM imaging.

### 2.8. Silk-CBD Assembly Model

**Figure 11:**
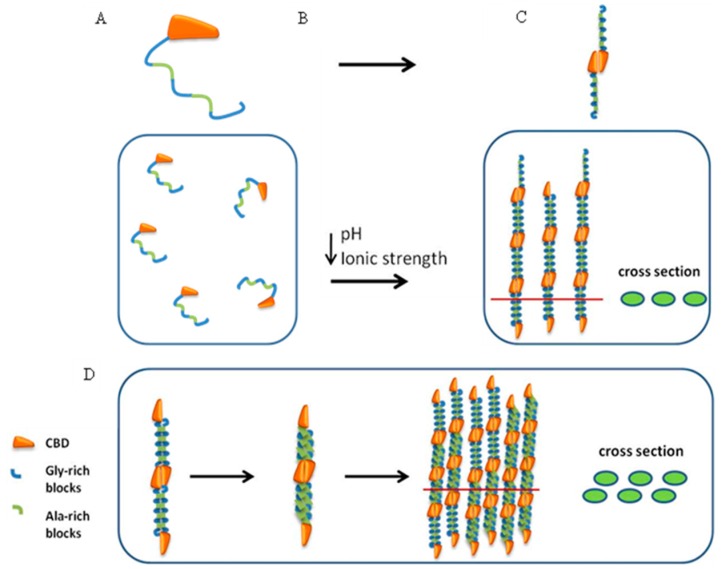
Silk-CBD assembly model. (**A**) silk-CBD in solution; (**B**) Concentration and dialysis of the silk-CBD protein solution leads to protein dimer formation (**C**); and (**D**) Sonication of silk-CBD exposes hydrophobic domains, which allow for further interactions, creating higher molecular assemblies.

**Figure 12:**
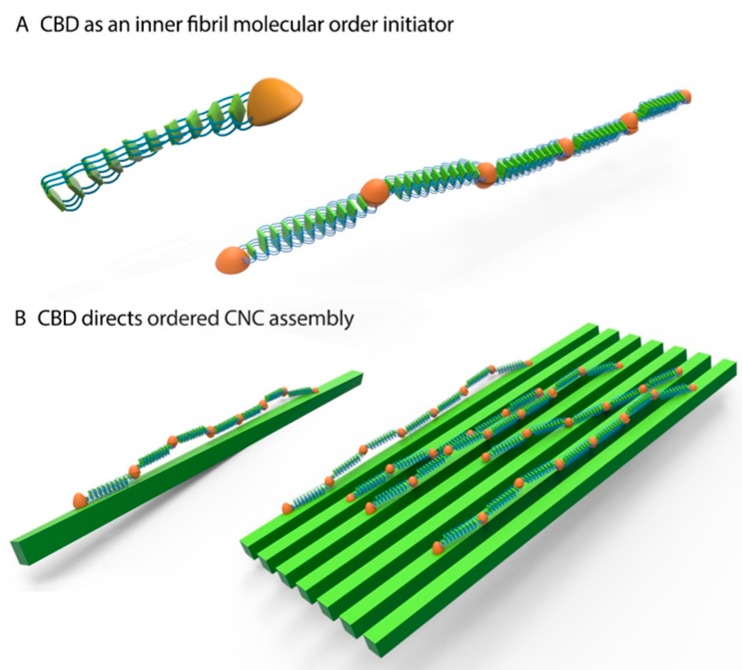
Schematic illustration of spider silk-CNC composite. CBD drives spider silk molecular ordering in two levels. (**A**) CBD’s ability to form dimers encourages spider silk alignment; and (**B**) CBD’s ability to bind CNCs drives directional alignment of silk-CBD-CNC composites.

Based on the gel filtration, DLS, SAXS and cryo-TEM findings, we suggest that silk-CBD forms dimers after solution concentration, which align to form liquid crystals (Figure 11C). After sonication, the hydrophobic spider silk protein domains are more prone to engage in further interactions, leading to higher molecular assemblies (Figure 11D). This molecular order likely effects the morphology and thermal properties of the silk-CBD-CNC composites, for instance by driving CNC alignment (see the mechanism presented in Figure 12).

## 3. Material and Methods

CNCs produced from the sulfuric acid hydrolysis were provided by Melodea Ltd. (Rehovot, Israel). Standard biochemical reagents and Sigmacote were purchased from Sigma-Aldrich (St. Louis, MO, USA), unless indicated otherwise.

### 3.1. Design and Expression of the Recombinant Spider Silk Proteins

A synthetic 15-monomer-long dragline spider silk gene was designed from a monomer consensus derived from the native sequence of MaSp1 of *Nephila clavipes* (Accession P19837) and then cloned into a pET30a vector, as previously described [45]. In order to produce the spider silk-CBD fusion gene, SpeI and XhoI restriction sites were introduced to the 5′ and 3′ of the CBD gene (Accession M73817.1), respectively, via PCR, using the following primers: CBDSpeI: 5′-GACTAGTATGGCAGCGACATCATCAATGTC-3′ and CBDXhoI: 5′-CTCGAGATCAAATGTTGCAGAAGTAGGATTAATTATTG-3′. The SpeI-CBD-XhoI construct was then fused to the 3′ of the silk gene in the pET30a-silk vector. The pET30a-silk and pET30a-silk-CBD vectors were transformed into BL21 (DE3) cells (Novagen, EMD Chemicals Inc., San Diego, CA, USA). Cells were cultivated in LB broth at 37 °C. Protein expression was induced by the addition of 1 mM IPTG (Fisher Scientific, Hampton, NH, USA) when the optical density (OD) at 600 nm (OD600) was between 0.6 and 0.9. After approximately 4 h of protein expression, the cells were harvested by centrifugation (10,000× *g* for 20 min at 4 °C).

### 3.2. Purification of 6H-Silk and 6H-Silk-CBD

Bacterial pellets of 400 mL culture were re-suspended in a 5 mL solution of 20 mM NaHPO_4_, 0.5 M NaCl, 10 mM imidazole plus Complete™ protease inhibitor (Roche Diagnostics, Mannheim, Germany) and sonicated on ice for several minutes in pulsed bursts. The soluble and insoluble pellets were separated by centrifugation at 15,000 rpm for 10 min, at 4 °C. The soluble fraction of the proteins was filtered using a 0.45 μm syringe filter for faster protein liquid chromatography (FPLC) (GE, Uppsala, Sweden) purification on a 5 mL HisTrap™ high Performance Ni-NTA column (GE, Uppsala Sweden), pre-equilibrated according to the user manual.

### 3.3. Quantitative Cellulose Binding Reversibility Assay for CBD, Silk and Silk-CBD

A binding reversibility assay was performed, as previously described [28]. CBD, silk, and silk-CBD protein concentrations ranging from 200 μg/mL to 1.2 mg/mL were allowed to adsorb onto 30 mg of prewashed cellulose (Sigmacell 20) for 30 min at 25 °C. After the protein’ adsorption to cellulose, a series of dilutions was carried out for the analysis of the desorbed protein in the following manner: the most concentrated protein-cellulose mixture (1.2 mg/mL protein + 30 mg cellulose) was diluted to final protein concentrations ranging from 1.2 mg/mL to 200 μg/mL, followed by an additional 30 min of mixing. After centrifugation of the diluted test tubes at 13,000× *g* for 10 min, the concentration of the bound protein was assayed using the Lowry method; the NaOH in the Lowry A solution elutes the bound proteins from the cellulose pellet.

### 3.4. Production of Composite Silk/Silk-CBD and CNC Sponges

The purified silk and silk-CBD proteins were dialyzed against water (dialysis ratio of 1:100) for 18 h, during which, the water was changed four times. After dialysis, the protein aqueous solution was concentrated to 6 wt. % using Vivaspin ultrafiltration (VS2001, 10,000 MWCO, Vivaproducts Inc., Littleton, CO, USA). The concentrated protein solution was mixed with 2.5% *w*/*w* CNC to yield the desired silk:CNC ratio. The mixture was then sonicated and poured into a Teflon mold. The mold was placed at ambient room temperature for 1 h and then frozen in a −20 °C freezer. The protein-CNC solution was then freeze-dried to generate a sponge.

### 3.5. CNC and Silk-CBD-CNC Films Preparation

Silk-CBD protein after dialysis and concentration was mixed into CNC suspensions (2.5 wt. %). Concentrated silk-CBD protein solution was mixed with 2.5% *w*/*w* CNC to yield the desired silk-CBD: CNC ratio by weight. The mixture was then sonicated and 15 mL of silk-CBD-CNC suspensions were cast onto a Sigmacote^®^ treated glass substrates. The silk-CBD-CNC suspensions were dried for 48 h under ambient conditions until a dried film was achieved.

### 3.6. Scanning Electron Microscopy (SEM)

Samples of the sponges and film were mounted on a metal stub and coated with gold (the coating thickness was 5 nm) using an Extra High Resolution Scanning Electron Microsopy Magellan™ 400L (FEI, Eindhoven, The Netherlands).

### 3.7. Polarized Light Microscopy (POM)

In order to investigate films orientation an LC-PolScope system (Cambridge Research and Instruments, Woburn, MA, USA) mounted onto a Nikon Eclipse 80i was used. The LC-PolScope image-processing system has the ability to quantify retardance/birefringence, and indicate molecular orientation. The molecular orientation is proportional to the angular shift in polarized light that occurs as it passes through a birefringent sample. Abrio 2.2 software was used for image acquisition and analysis, background and specimen images were captured under identical conditions.

### 3.8. Differential Scanning Calorimeter (DSC) Analysis

A TA Instruments (New Castle, DE, USA) Q100 differential scanning calorimeter (DSC), with purged dry nitrogen gas flow (50 mL/min), and equipped with a refrigerated cooling system, was used to study the thermal properties of the samples (~5 mg each in Al pan)Heat flow and temperature were calibrated with indium. Temperature-modulated DSC (TMDSC) measurements were performed at a heating rate of 2 K/min, with a modulation period of 60 s and temperature amplitude of 0.318 K. Aluminum and sapphire reference standards were used for calibration of the heat capacity. In TMDSC, the “reversing heat capacity”, which represents a reversed heat effect within the temperature range of the modulation, was measured and calculated.

### 3.9. Gel Filtration

A prepacked Superdex™ 200 10/300 GL column was obtained from Amersham Pharmacia Biotech (Piscataway, NJ, USA) and calibrated using a molecular weight markers kit, (product no. MW-GF-1000, Sigma-Aldrich, St. Louis, MO, USA). Markers included β-amylase (200 kDa), albumin (66 kDa), carbonic anhydrase (29 kDa). Samples were applied to the column using 20 mM sodium phosphate buffer, pH 7.4, as a running buffer, at a flow rate of 0.3 mL/min. Protein was detected with a UV detector, at a wavelength of 280 nm. Samples were collected at the column exit every 0.5 min. The molecular weights of the respective samples were calculated using the linear column calibration curve.

### 3.10. Dynamic Light Scattering (DLS)

Hydrodynamic radius measurements were performed with a DynaPro MSTC800 instrument (Protein Solutions, Inc., Charlottesville, VA, USA). Fresh protein stock solutions were diluted in DDW to a final concentration of 2 mg/mL. Samples were centrifuged at 13,000× *g* for 10 min, to remove insoluble particles. A 1 mL aliquot of each sample was placed directly into a quartz cuvette, gently mixed and analyzed every 10 s, at 25 °C. The estimated hydrodynamic radii were calculated using Dynamic V6 data analysis software.

### 3.11. Small Angle X-ray Scattering (SAXS)

Sonicated and un-sonicated protein samples (6% *w*/*v*) were transferred into quartz capillary tubes, which were then flame-sealed. The sealed capillary tubes were centrifuged at 6000× *g*, using a Sigma 1-15PK centrifuge equipped with a capillary rotor. Solution small angle X-ray scattering measurements were performed using a state-of-the-art in-house setup, described elsewhere [46]. Scattering images were radially integrated and the form and the structure factors were analyzed using the X+ program [47]. X+ is a user-friendly, comprehensive, computationally accelerated software program for the analysis of radially integrated signals of solution X-ray scattering from supramolecular self-assembled structures. This system can address both the form and structure factor contributions to the signal.

### 3.12. Cryo-Transmision Electrom Microscopy (TEM)

Samples of silk and silk-CBD (4 μL) were applied to a 300-mesh copper grid coated with lacey carbon (Structure Probe, Inc. Supplies, West Chester, PA, USA). Samples were blotted in a controlled environment held at 25 °C and 100% relative humidity, and then plunged into liquid ethane using a CEVS plunger. Specimens were equilibrated at −178 °C in a Tecnai F20 transmission electron microscope operated at 200 kV, using a Gatan 626 cooling holder and transfer station, with a TVIPS F415 CCD digital camera at the Department of Chemical esearch Support, The Weizmann Institute of Science, Rehovot 76100, Israel.

## 4. Conclusions

A unique approach to harness the structural alignment and cross-bridging of spider silk proteins to CNCs for the production of silk-CNC composite materials was used; this new approach involves the concentration and sonication of silk-CBD, which seems to influence the alignment and morphology of the composite. We propose that the higher molecular order observed in these composites is due to (1) CBD dimerization (and higher order assemblies); and (2) the binding of CBD to CNCs. These new composites may be useful in a variety of applications, including medical ones. In the model depicted in Figure 12, CBD induces spider silk molecular order at two levels. First, the ability of CBD to form dimers and to mimic the nonrepetitive spider silk terminal function encourages molecular ordering towards the formation of aligned nano-silk fibers. Second, at a higher level, CBD specifically mediates silk-CNC interactions, guiding the formation of structural and ordered composite materials. Further work is currently underway toward exploring the mechanical properties of these composites, and whether the differences seen here, e.g., morphology and thermal properties, translate into improved/altered mechanical properties.

## Figures and Tables

**Table 1:** Glass transition and degradation temperatures of silk and silk-CBD sponges, as determined from TMDSC analysis.

Sample	Glass Transition Temperature (*Tg* °C)	Degradation Temperature (°C)
100% silk	140.5	283
100% silk-CBD	172	279
75% silk 25% CNC	138	255
75% silk-CBD 25% CNC	174	269
25% silk 75% CNC	163	231
25% silk-CBD 75% CNC	178	237

**Table 2:** Summary of DLS results of silk and silk-CBD samples before and after sonication (*n* = 10, average ± standard deviation).

	Silk	Sonicated Silk		Silk-CBD		Sonicated Silk-CBD	
**diameter (nm)**	2.78 ± 0.04	3.8 ± 0.11	825 ± 401	55 ± 36	259 ± 30	96 ± 8	2048 ± 691
**% volume**	100	99.9	0.1	32	68	64	36
